# Eco-anxiety in children: A scoping review of the mental health impacts of the awareness of climate change

**DOI:** 10.3389/fpsyg.2022.872544

**Published:** 2022-07-25

**Authors:** Terra Léger-Goodes, Catherine Malboeuf-Hurtubise, Trinity Mastine, Mélissa Généreux, Pier-Olivier Paradis, Chantal Camden

**Affiliations:** ^1^Faculty of Medicine and Health Sciences, Université de Sherbrooke, Sherbrooke, QC, Canada; ^2^Department of Psychology, Bishop's University, Sherbrooke, QC, Canada; ^3^Centre de Recherche du Centre Hospitalier Universitaire de Sherbrooke (CRCHUS), Sherbrooke, QC, Canada; ^4^School of Communication Sciences and Disorders, McGill University, Montreal, QC, Canada; ^5^Institut universitaire de première ligne en santé et services sociaux (IUPLSSS) du Centre intégré universitaire de santé et services sociaux (CIUSSS) de l'Estrie, Sherbrooke, QC, Canada; ^6^Department of Psychology, Université de Sherbrooke, Sherbrooke, QC, Canada

**Keywords:** eco-anxiety, children, youth, mental health, climate change, scoping review

## Abstract

**Background:**

Youth are increasingly aware of the negative effects of climate change on the planet and human health, but this knowledge can often come with significant affective responses, such as psychological distress, anger, or despair. Experiencing major “negative” emotions, like worry, guilt, and hopelessness in anticipation of climate change has been identified with the term eco-anxiety. Emerging literature focuses on adults' experience; however, little is known about the ways in which children and youth experience eco-anxiety.

**Objectives:**

The aim of this review was to: (1) identify the available evidence on the topic of eco-anxiety in children, (2) clarify the mental health consequences brought by the awareness of climate change in this population, and (3) identify knowledge gaps in the literature and considerations for future research.

**Methods:**

Given that the research on the topic of eco-anxiety in children is limited, that there are very few randomized controlled trials, and that the goal is not to analyze individual studies in-depth, a scoping review was used. Keywords pertaining to the themes of eco-anxiety, climate change and children (aged < 18 years) were used as search terms in five databases. Journal articles using qualitative and quantitative methods, as well as gray literature were examined by two independent reviewers. A descriptive-analytical method was used to chart the data that emerged from the literature. Eighteen articles were considered in the final analysis.

**Results:**

Evidence confirms that children experience affective responses and eco-anxiety in reaction to then awareness of climate change. Mental health outcomes include depression, anxiety, and extreme emotions like sadness, anger, and fear. Youth from vulnerable communities, like indigenous communities, or those who have strong ties to the land are often identified as being emotionally impacted by climate change. The literature analyzed also describes how children and youth are coping with eco-anxiety, including maladaptive (e.g., denial) and adaptive responses (such as constructive hope, used as a positive coping mechanism). Preliminary considerations for parents, teachers and educators, mental health care providers, school systems, adults and people of power include adding age-appropriate climate education to the school curriculum, considering youth's emotions, and promoting healthy coping through empowerment. Important gaps exist in the definition of eco-anxiety in youth, as various characterizations of this emerging concept are found across articles.

## Introduction

Although climate change has been a subject of interest since the 1960s (for example, see Flohn, 1961; Benton, 1970), it has undeniably become the challenge of the 21^st^ century. The Intergovernmental Panel for Climate Change (IPCC), which has published scientific assessments about climate change since 1990, issued a report presenting different scenarios for the future, outlining the inevitable consequences humans will face in the next years (Masson-Delmotte et al., 2021). In all these scenarios, it is unavoidable that temperatures will reach an increase of 1.5 degrees Celsius by 2040 (with 1850–1900 being the baseline). As such, there is scientific consensus of anthropogenic (i.e., human-made) climate change (Costello et al., 2009; Hoegh-Guldberg et al., 2019; Masson-Delmotte et al., 2021). This phenomenon not only affects biodiversity in general, but it also has significant physical impacts on humans (Hoegh-Guldberg et al., 2019; Masson-Delmotte et al., 2021). For example, researchers have found that pollution can cause acute respiratory problems (Doherty et al., 2017; Orru et al., 2017), some even linking the increase in viral infections to the destruction of ecosystems (Woolhouse, 2002; Everard et al., 2020).

The Media and Climate Change Observatory (MeCCO) published a special issue on the particular attention that was brought to the topic of climate change in 2019 (Nacu-Schmidt et al., 2020). They highlighted how the media devoted much attention to the increasing number of fires around the world, the rising sea levels, and the United Nations climate talks. These events from 2019 also coincided with the global youth-led climate strikes and the appearance of Greta Thunberg as a public personality. Indeed, people around the world were becoming worried about the future of the planet and the increasingly frequent extreme weather events. As there was a clear consensus of the physical effects of climate change (on humans, biodiversity, and the Earth), people were realizing that there were also emerging mental health impacts (Arcanjo, 2019; Ballew et al., 2019). Furthermore, in the midst of the COVID-19 pandemic, people are facing two major crises which both take a toll on mental health (Généreux et al., 2020; Rosen, 2020; Samji et al., 2021). Not only are youth particularly affected by restrictive measures (quarantine, school closures), but they are also concerned for their future when they integrate messages of emergency and crises relative to climate change (Pickard, 2021; Romeu, 2021).

## Mental health and climate change

Berry et al. (2010) propose three categories of mental health impacts of climate change: direct, indirect, and vicarious. Most research has focused on the direct impacts of climate change on mental health, which are those that happen after experiencing an extreme weather event such as a flood, an earthquake, or a hurricane. These major life disruptions can lead to post-traumatic stress disorder (PTSD), depression disorders, anxiety disorders, substance use disorders, and suicidal thoughts (Berry et al., 2010; Hayes et al., 2018; Cianconi et al., 2020). Indirect impacts of climate change can also affect mental health through consequences on the economy, migration, damage to physical and social infrastructure, food and water shortages, and conflict; all of which have been linked to stress, grief, anxiety and depression (Akresh, 2016; Hayes et al., 2018). However, even without experiencing the direct or indirect effects of climate change, many feel distress simply by being aware of the global environmental crisis (Pihkala, 2018).

The focus of this review will be on these *vicarious* reactions, or the emotional and affective impacts of the awareness of climate change lived through knowledge about the issue. In other words, witnessing the effects of climate change through the media and other information sources without experiencing it firsthand can also have an impact on mental health. There is much less research on this form of vicarious reaction to climate change, but scholars are starting to report that people are feeling overwhelmed by the situation, leading to panic attacks, insomnia, or obsessive thinking (Clayton et al., 2017; Usher et al., 2019). Knowing about climate change and its consequences can bring upon many emotions such as guilt, sadness, and anger, which all make up *eco-anxiety* (Pihkala, 2020). The American Psychological Association (APA) recognizes eco-anxiety as a “chronic fear of environmental doom” (Clayton et al., 2017).

Researchers have found that experiencing low levels of anxiety and emotions in the face of climate change is a normal response to a stressful reality (Reser and Swim, 2011; Verplanken and Roy, 2013; Clayton et al., 2017; Scheirich, 2020). As such, there is no mental health diagnosis of eco-anxiety, and it is not considered a pathological problem. However, some people who are experiencing eco-anxiety will feel genuine distress that can limit their daily activities and lead to serious depressive and anxious symptoms (Jones et al., 2012; Doherty, 2018; Pihkala, 2018; Kaplan, 2020; Rosen, 2020). Thus, some researchers are now suggesting that the concept of eco-anxiety may be placed on a spectrum: on one end, these strong emotions may lead to action and mobilization, empowering people to change their habits and help the planet; on the other end, eco-anxiety may lead to a debilitating paralysis when facing the immensity of the problem (Wolf and Moser, 2011; Wolfe and Tubi, 2019; Pihkala, 2020). In short, as knowledge about climate change can lead to an increase in pro-environmental behaviors, it can also lead to paralyzing anxiety and denial (Albrecht et al., 2007). People may move along this spectrum depending on many factors, including their emotional availability, social support network, and the world's global situation (Berry et al., 2010; Clayton et al., 2017; Hayes et al., 2019). This broader definition of eco-anxiety acknowledges that strong emotional reactions to the current climate crisis are normal and could lead to pro-environmental behaviors (Searle and Gow, 2010). Whereas, an overly restricted definition of eco-anxiety might lead to it being pathologizing when people could use the emotions elicited by eco-anxiety positively as a motivation for taking action and learn adaptive coping strategies (Verlie, 2019). This is especially important since the problem of climate change is unlikely to disappear in the next few years (Masson-Delmotte et al., 2021).

Some scholars have suggested that the term eco-anxiety may not be the most suited to describe this phenomenon since the name seems to refer solely to anxiety when its definition encompasses many other emotions (Arcanjo, 2019; Huizen, 2019; Ojala, 2019; Pihkala, 2020; Raypole and Legg, 2020). Nonetheless, given the popularity of the term, this review employs the broad definition of eco-anxiety, referring to this key concept as any significant emotional response to the awareness of climate change.

Literature is rapidly emerging on eco-anxiety in adults, but very little remains known about how younger people and children experience the awareness of climate change. Children around the world are growing up in an uncertain world where messages of “doom and gloom” about climate change often dominate the public discourse and media (Engelhaupt, 2017). In Australia, a survey of 600 children between 10 and 14 years-old children revealed that “44% of children are worried about the future impact of climate change” and “one quarter of children worry that the world will end before they get older” [(Tucci et al., 2007). p. 13]. Learning about climate change without acquiring the tools to cope with the emotions that accompany this knowledge may lead to hopelessness and denial (Ojala, 2012b). Nonetheless, general knowledge about climate change amongst younger people seems low, but their level of concern and anxiety is high (Erkal et al., 2012; Corner et al., 2015). The mental health consequences of eco-anxiety in children are not yet well understood. Thus, a review of the current state of the literature may help to understand the extent of the academic knowledge on this subject.

### Objectives

Eco-anxiety may very well be a public health challenge in the years to come; thus, it is important to continually stay up to date with the findings. Scoping reviews are a relevant methodological tool when the research on the topic is rapidly growing, that there are very few randomized controlled trials, and that the goal is not to analyze individual studies in-depth (Munn et al., 2018). As such, the objective of this study was to conduct a scoping review of the relevant articles on the subject of eco-anxiety in children and youth. The broad guiding research question was: What is the nature of the evidence on eco-anxiety in youth and children? Although the phenomenon of eco-anxiety is gaining popularity in research, there is a lack of understanding of the vicarious psychological impacts of climate change in youth and children (Cunsolo et al., 2020). The aims of this study were to: (1) explore the terminology used to describe eco-anxiety in youth and children specifically, (2) look at the evidence of eco-anxiety in this population, and (3) identify the knowledge gaps in this research topic and highlight specific considerations in relation to future research. An initial search of the literature revealed that evidence on the topic of eco-anxiety focused on initial theories and many suggested specific recommendations in relation to children coping with climate change awareness. Although providing recommendations may be precocious, given that eco-anxiety in children is a rapidly emerging topic, it is important to focus on ways to support mental health. Nonetheless, one must bear in mind that these recommendations are based on knowledge that is preliminary in nature, consequently, they must be analyzed with caution. Hence, these recommendations that emerged are regarded as specific considerations for certain actors in children's lives that should guide future research.

## Methods

### Protocol

The study protocol for this review was developed using the methodological framework suggested by Arksey and O'Malley (2005), which was further refined by Levac et al. (2010) and was guided by the Preferred Reporting Items for Systematic Reviews and Meta-Analyses for scoping reviews (PRISMA-ScR) statement (Tricco et al., 2018).

### Inclusion and exclusion criteria

The present review considered studies published between January 1^st^, 2000, and March 3^rd^, 2021, in English and in French, from around the world. The lower date limit was selected given that eco-anxiety and the urgency of the climate crisis are topics that have gained focus since the beginning of the 21^st^ century (Pihkala, 2020). The population of interest for this scoping study were children and youth. The United Nations Convention on the Rights of the Child definition of children was used to identify our population as “human beings below the age of 18” (United Nations., 1989).

We operationalized our review based on the work from a prominent figure in the field, Pihkala, who conducted a systematic analysis to clarify the nature of the phenomena of eco-anxiety, including articles from social and political sciences, existential psychology, psychodynamic perspectives, and other anxiety theories (Pihkala, 2020). Indeed, they define the concept of eco-anxiety as “a general term for difficult feelings because of the ecological crisis” (p. 14). Common emotional responses include anger, guilt, sadness, and hopelessness (Clayton et al., 2017; Pihkala, 2018). Similarly, we broadly defined the concept of eco-anxiety as any strong emotion pertaining to the awareness of climate change. Keywords included hopelessness, solastalgia, eco-phobia, eco-depression, despair, eco-angst, and eco-guilt. These keywords were found in literature on the topic of eco-anxiety pertaining to adults, and in the review by Pihkala (2020).

The exclusion criteria were selected based on the chosen definition of eco-anxiety: an anticipatory emotional response to climate change and on the chosen population of children. Thus, the articles included needed to (1) involve children, (2) mention mental health impacts of climate change, and (3) these impacts needed to be in response to the awareness of climate change. As such, excluded articles that did not meet criteria were those that only involved people above 18 years old; spoke of physical, economical, or social impacts without mentioning mental health; or only touched on the effects from after a climate-related natural crisis such as a tornado or a tsunami.

### Types of sources

Given that the aim of a scoping study is not to evaluate the quality of the evidence, but to summarize the evidence and make recommendations, quantitative, qualitative, observational, mixed methods, and review studies, as well as opinion papers, news articles and dissertation theses were included. Research protocols and scale elaborations were not included, as they did not inform on the nature of the evidence.

### Search strategy

To help identify keywords and index terms, an initial search in APA PsycInfo was performed with an information specialist. This informed the full search strategy. The information sources were selected based on the interdisciplinary nature of the concept of eco-anxiety, with a particular interest for psychology and education sources. The selected electronic databases were APA PsycInfo, Education source, ERIC, GreenFILE, MEDLINE, Psychology and Behavioral Sciences Collection, SocINDEX, Sage Journals and ProQuest. Furthermore, gray literature (non-published materials) was identified through a simple Google search (i.e., not scholar), using the first five pages of the results. Materials in English and French were included, given these were the languages understood by the reviewers. The final search strategy for the APA PsycInfo database can be found in Appendix A. Key articles' bibliographies (Ojala, 2012a; Strife, 2012) were analyzed to identify further relevant literature.

### Study selection

**Figure 1:**
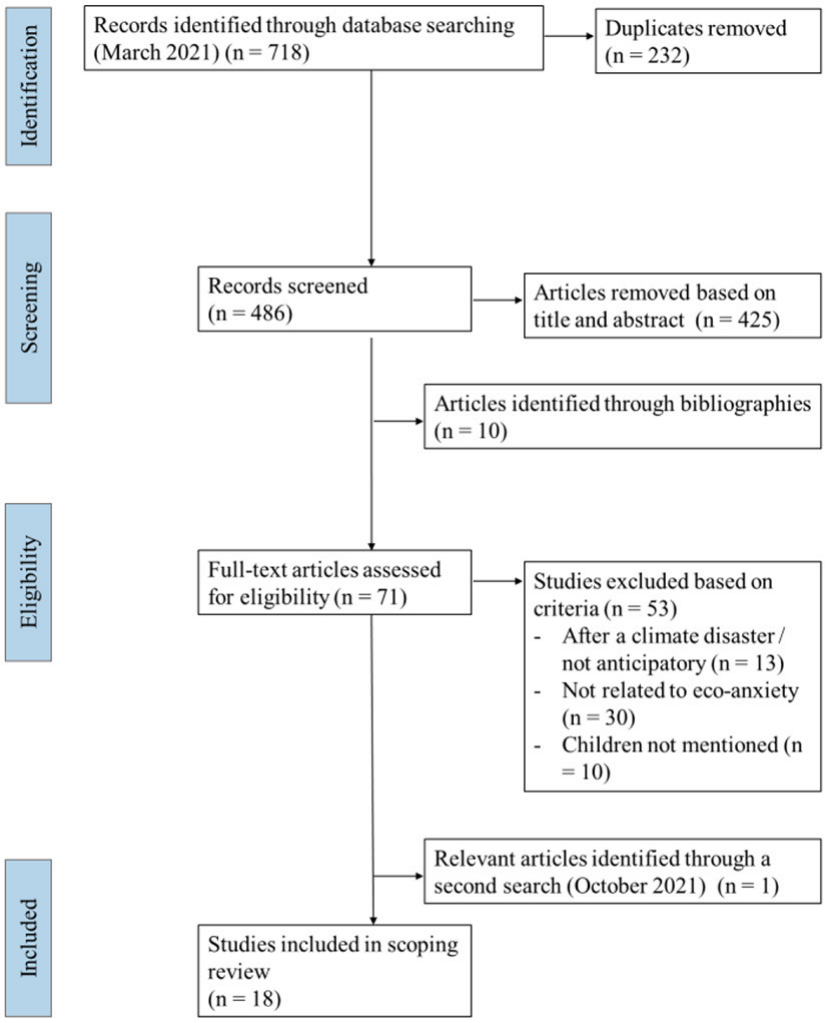
Preferred reporting items for systematic reviews and meta-analyses extension for scoping reviews (Prisma-ScR) flow diagram.

A total of 718 sources were identified in the full search stage and 486 sources remained after duplicates were removed. Identified articles were saved into Mendeley (version 1.19.8) and uploaded to Covidence (version v2532 1ea49715) for two reviewers to independently screen the articles. The inclusion and exclusion criteria were specified in the software and were used in the level 1 and level 2 screenings. The two independent reviewers first screened the titles and abstracts, and the percentage of agreement was 81% for this step. Next, the same two reviewers independently screened the full text (percentage of agreement = 82%). For each step, the reviewers met to discuss any conflicts in their decisions. If needed, a third reviewer was included to help decide on inclusion. Please see Figure 1 for the flow diagram based on PRISMA (Tricco et al., 2018). A total of 17 articles remained.

Given that eco-anxiety is an emerging area of research with an increase in publications in the past year, a second search was performed using the same keywords and databases, with the aim of including any newly published article between March 2021 and October 23rd, 2021. A total of 375 articles were identified, 13 titles and abstracts were screened, and one met the full inclusion criteria.

### Data extraction

Data was extracted from the Covidence software and charted in a pre-defined charting form (see Appendix B) that was further refined. Two independent reviewers charted the relevant information into categories, including author/year, country of publication, research method used, objectives of the study, definition of eco-anxiety, and general impacts of climate change awareness on children's mental health.

## Results

This scoping review considered 18 articles that met all inclusion/exclusion criteria. The 18 articles included in the scoping review are identified in the reference list using a “*” symbol. The data was extracted into a table, exploring the definitions of eco-anxiety, the mental health impacts of climate change awareness on children's mental health, vulnerability or protective factors, and key findings by the authors. Research gaps were then identified and discussed between the researchers.

### Study characteristics

All selected studies were published after 2002, with 60% being published after 2016. Half of the articles were from the United States, and the most common research method used was a descriptive design by means of a cross sectional survey (44%) and none of the articles included inferential statistics. A few employed a review type of analysis of evidence (22%), but no formal guide was used (i.e., not systematic, not scoping, etc.). The most common fields of research of the first author were education (39%) and psychology (28%). Two articles were from non-academic sources (newspapers). The study characteristics can all be found in Table 1, and the methodological information (methods, sample size and age) for each article can be found in Table 2.

**Table 1:** Study characteristics.

**Study characteristics** **(*****N*****= 18)**		**Count (%)**
**Year of publication**		
	2002-2005	2 (11.1%)
	2007	1 (5.6%)
	2012-2013	4 (22.2%)
	2016-2019	4 (22.2%)
	2020	6 (33%)
	2021	1 (5.6%)
**Country**		
	United States	9 (50%)
	Sweden	3 (16.7%)
	England	1 (5.6%)
	Finland	1 (5.6%)
	Australia	1 (5.6%)
	Australia and Canada	1 (5.6%)
	Canada and Taiwan	1 (5.6%)
	Mixed	1 (5.6%)
**Research method**		
	Descriptive cross-sectional survey (quantitative)	8 (44.4%)
	Review	4 (22.2%)
	Interviews (qualitative)	2 (11.1%)
	Newspaper article	2 (11.1%)
	Observational (qualitative)	1 (5.6%)
	Analysis of written letters (qualitative)	1 (5.6%)
**Field**		
	Education	7 (38.9%)
	Psychology	5 (27.7%)
	Environmentalism	3 (16.7%)
	Journalism	2 (11.1%)
	Nursing	1 (5.6%)

**Table 2:** Methodological information arranged per author.

**Reference**	**Methods**	**Sample size**	**Sample age**
Hickman et al. (2021)	Cross sectional survey	*n* = 10 000	16–25 years old
Chalupka et al. (2020)	General review (no specific method)	None mentioned	“Children”
Pinto and Grove-White (2020)	Observational	One primary school (sample size not further specified)	Primary school aged children (5–12 years old)
Plautz (2020)	Newspaper article	None mentioned	12–25 years old
Ratinen and Uusiautti (2020)	Cross-sectional survey (descriptive design)	950	11–17 years old (M = 13.6)
Taylor and Murray (2020)	Newspaper article	None mentioned	“Children” and “Youth”
Zummo et al. (2020)	Qualitative analysis of letters	*n* = 350	12–18 years old
Li and Monroe (2019)	Cross-sectional survey (descriptive design)	*n* = 728	13–18 years old (9–12^th^ graders)
Burke et al. (2018)	General review (no specific method)	None mentioned	“Children”
Boggs et al. (2016).	General review (no specific method)	None mentioned	“Children”
Stevenson and Peterson (2016)	Cross-sectional survey (descriptive design)	*n* = 1486	11–15 years old
Ojala (2013)	Cross-sectional survey (descriptive design)	*n* = 321	M = 17.2 years old
Ojala (2012a)	Cross-sectional survey (descriptive design)	Late childhood (*n* = 90) Adolescents (*n* = 146)	Late childhood (M = 11.7 years old) Adolescents (M = 16.4 years old)
Ojala (2012b)	Cross-sectional survey (descriptive design)	*n =* 293	12 years old
Strife (2012)	Semi-structured qualitative interviews	*n* = 50	10–12 years old
Sobel (2007)	General review (no specific method)	None mentioned	“Children”
Nagel (2005)	Phenomenological interviews	*n* = 40	7^th^ grade (12–13 years old)
Huang and Yore (2005)	Cross-sectional survey (descriptive design)	*n* = 761	5^th^ grade (10–11 years old)

### Terminology used

Our first objective was to explore the terminology used in the selected articles around the theme of eco-anxiety in literature focused on children. The term eco-anxiety was only employed in three articles (16%) (Pinto and Grove-White, 2020; Plautz, 2020; Hickman et al., 2021). Authors Pinto and Grove-White (2020) started their article with the sentence “Eco-anxiety is on the rise and is affecting children of all ages, including primary children” (p. 252), but they did not define the term. The article they cited while using the term eco-anxiety was a BBC Newsround survey that found that 70% of 8- to 16-year-olds report feeling worried about the state of the planet (Atherton, 2020). As such, it could be extrapolated that these authors perceived eco-anxiety as significant worry about the state of the planet because of climate change. In their news article published in the Washington Post, Plautz (2020) also used the term without explicitly defining it. They referred to eco-anxiety as a type of anxiety felt (by children and adolescents) in response to climate change, and significant worry about the planet. For their part, Hickman et al. (2021) defined eco-anxiety as “distress relating to the climate and ecological crises” (p. 3). They also included many different emotions as being involved in eco-anxiety, including “worry, fear, anger, grief, despair, guilt, and shame, as well as hope.” (p. 3) In general, authors from these three articles seemed to agree that worry is a key component of eco-anxiety in children.

Other terms related to eco-anxiety were also used. These included ecophobia, which was referred to as being “fearful of environmental problems” [(Ratinen and Uusiautti, 2020). p. 11], a sense of “ennui and helplessness” caused by the “overwhelmingness of environmental problems” [(Sobel, 2007). p. 17], and a “broad fear of environmental deterioration and environmental problems” [(Strife, 2012). p. 37]. Li and Monroe (2019) use the terms environmental grief and eco-despair without defining them. The term climate anxiety was used by authors to refer to as “distress relating to the climate and ecological crises” [(Hickman et al., 2021). p. 3] and as “the fear that the current system is pushing the Earth beyond its ecological limits” [(Taylor and Murray, 2020). p. 3].

### Evidence of eco-anxiety in children

Our second objective was to identify the evidence of eco-anxiety in children. Although the term eco-anxiety was not frequently used, many of the articles mentioned the emotions reported by children and youth that were included in the definition of eco-anxiety. To identify the emotions and psychological states reported, a content analysis was used (Erlingsson and Brysiewicz, 2017). Each passage was coded to indicate emotions felt by children or youth. The authors of the included articles mentioned that children and youth feel fear, anger, hopelessness, and sadness as they become aware of climate change and its consequences. However, worry and hope were the two emotions that were most reported in the selected articles. For the occurrences coded as being an emotional reaction of children to the awareness of climate change, please see Table 3.

**Table 3:** Coding frequency for each emotion/state in all the selected articles.

**Emotion/state**	**Number of relevant codes** **(% of codes)**	**Number of articles that mention (%)**
Worry	88 (26.9%)	14 (78%)
Hope	68 (20.8%)	12 (67%)
Fear	47 (14.4%)	12 (67%)
Anxiety	43 (13.1%)	15 (83%)
Anger	23 (7%)	12 (67%)
Despair	16 (4.9%)	7 (39%)
Sadness	14 (4.3%)	10 (39%)
Hopelessness	14 (4.3%)	9 (50%)
Guilt	11 (3.4%)	5 (28%)
Depression	3 (0.9%)	2 (11%)

Furthermore, data was charted to describe how each article interpreted the impacts of climate change awareness on children's mental health, as well as the vulnerability and protective factors (see Table 4). Many indicators suggested that children are experiencing eco-anxiety. Several authors indicated that children are feeling worried in their daily lives. For some, this can manifest as a concern for children and people from other countries who are already experiencing the impacts of climate change (Burke et al., 2018; Chalupka et al., 2020; Taylor and Murray, 2020). For others, this worry pertained to their own future, anticipating a significant loss of biodiversity, an increase in pollution, and perhaps even the end of the world in their own lifetime (Huang and Yore, 2005; Nagel, 2005; Strife, 2012; Ojala, 2013; Burke et al., 2018; Chalupka et al., 2020; Plautz, 2020; Ratinen and Uusiautti, 2020; Hickman et al., 2021). This considerable worry about the future of the planet and how the world will be when they grow up can lead to hopelessness and pessimism. This worry is also closely linked to emotions of fear for their future (Huang and Yore, 2005; Strife, 2012; Boggs et al., 2016; Burke et al., 2018; Pinto and Grove-White, 2020; Plautz, 2020; Zummo et al., 2020), anger that their generation must deal with this problem (Huang and Yore, 2005; Ojala, 2012a; Strife, 2012; Boggs et al., 2016), general states of anxiety (Ojala, 2012b; Boggs et al., 2016; Stevenson and Peterson, 2016; Burke et al., 2018; Pinto and Grove-White, 2020; Plautz, 2020; Ratinen and Uusiautti, 2020; Taylor and Murray, 2020) or even panic attacks (Plautz, 2020).

**Table 4:** Charted scoping review data from all 18 papers, arranged chronologically and by author.

**Authors**	**Impact of climate change awareness on children's mental health**	**Vulnerability factors**	**Protective factors**
Hickman et al. (2021)	A significant amount of youth and young adults feel very worried about climate change and report feeling afraid, sad, anxious, angry, powerless, helpless, and/or guilty. These strongly felt emotions impacted the daily lives of 45% of this sample.	Distress was higher when youth believed that government responses are inadequate.	Coping skills, validation, respect of their views/feeling heard, and agency to act.
Chalupka et al. (2020)	Children from “high-income developed nations” are familiar with the impacts of global climate change. This awareness causes significant concern and internal conflicts given their perceived privilege.	Children from indigenous and subsistence communities or that have strong ties to the land may experience more concern for the planet.	None mentioned.
Pinto and Grove-White (2020)	Information about climate change that is not adapted to children can lead them to feel scared and anxious about the situation. They need opportunities to deal with these emotions and act for the environment.	Fear without the possibility of action can lead to significant anxiety and feelings of hopelessness.	Primary schools supporting climate action (feelings of coherence between values/what is learnt and institution's choices).
Plautz (2020)	Climate change is an additional burden to children. Some children may have trouble coping with their daily stress combined to the awareness of climate change, potentially leading to hopelessness, and wondering why they should keep going to school. Some teenagers express fear for their future, panic, sadness, and injustice that their generation must deal with the problem of climate change and its consequences.	Being a child or an adolescent, as they become aware of the task ahead and the consequences of climate change, they will have to deal with in their lifetime, can make them experience significant distress and anxiety.	None mentioned.
Ratinen and Uusiautti (2020)	Students experience pessimism and doubt that the future will change to respond to climate change. This lack of hope can lead to depression and anxiety.	Girls have a higher sense of responsibility to mitigate climate change and they are less confident of the effectiveness of science than boys, making them more vulnerable to anxiety and pessimism.	Fostering constructive hope and optimism in children can help them face the challenge of climate change.
Taylor and Murray (2020)	Children are aware of global climate change at a very young age and can become stressed or anxious.	None mentioned.	Empowering children to take action for the environment can help them reduce their anxiety.
Zummo et al. (2020)	Discourses of doom that often taint the media coverage of climate change and can lead to children experiencing fear and anxiety, leading to inaction, loss of hope and a later focus on the negative.	None mentioned.	None mentioned.
Li and Monroe (2019)	Students' concern about climate change and hope are positively correlated. Being aware of and concerned about the environmental crisis leads to finding strategies to be more effective and cope with the emotions.	None mentioned.	Older girls had the highest levels of hopefulness in this sample.
Burke et al. (2018)	Many young people experience worry, fear, and anxiety about the consequences of climate change on their future lives. Children also express concern about the impacts of climate change on children who live in developing countries. Climate change awareness can also lead to despair, denial, and inaction, as they are unable to cope with the extent of the problem.	None mentioned.	Activism and implication in youth climate movements can help children manage their anxiety (action). Positive images of the future with achievable actions can help children build hope.
Boggs et al. (2016)	Children ask questions about their personal impact on the planet and wonder how they can make a difference given the size of the problem. As such, they can experience anxiety, fear, and anger.	None mentioned.	None mentioned.
Stevenson and Peterson (2016)	Concern about climate change may be a positive way to lead to action. It is particularly important that children feel concern in response to climate change because they will have to face the consequences of climate change during their lifetime and are particularly vulnerable to despair. As such, anxiety and worry in adolescents could be linked to critical thinking and engagement in solutions. Nonetheless, this mechanism is only at work if the concern and anxiety is accompanied by hope	Girls experience higher levels of concern.	Girls are also more hopeful.
Ojala (2013)	Children may experience worry, negative affects, and/or anxious and depressive feelings in response to their awareness of climate change.	Children who cope using problem-focused coping mechanisms may experience more negative affect.	Children who cope using meaning focused coping may experience more positive affect and higher life satisfaction.
Ojala (2012a).	Children and adolescents are worried and feel negative emotions about climate change. Emotions mentioned include guilt, anger, sadness, and hopelessness.	Adolescents are more vulnerable to experiencing pessimism.	Children and adolescents who cope using meaning focused coping feel less helpless.
Ojala (2012b).	Children seem to significantly worry about global climate change and many solely cope by searching for information about ways to solve the problem (problem-focused coping) which, when used to cope with an uncontrollable situation, can lead to lower mental wellbeing.	None mentioned.	Having a sense of purpose may reduce the feelings of worry in children. Using other coping mechanisms combined to problem-focused coping can reduce anxiety and negative emotions.
Strife (2012)	Children express sadness, fear, anger, pessimism, and feel overwhelmed by the awareness of climate change. Some children report crying, having nightmares, feeling extreme sadness and fear that the world might end, some fear for their own children, and other say they want to scream when hears about climate change.	None mentioned.	Trust in technology was associated to higher hope.
Sobel (2007)	Environmental education can lead children to feel overwhelmed and worried.	None mentioned.	When children think their behavior makes a difference (feeling of agency), they are less worried.
Nagel (2005)	Many students expressed concern, and children are susceptible to negative feelings because of environmental information they may get in the media. Some children experienced learned hopelessness (deterioration of the environment is at a point of no return), and this led to inaction and apathy (lack of interest and concern), action paralysis, and disempowerment.	None mentioned.	None mentioned.
Huang and Yore (2005)	Children express fear, anger, and worry about pollution and environmental problems.	None mentioned.	None mentioned.

However, there is also evidence that indicates that youth and children experience hope in the context of climate change awareness. Two studies found that worry and hope were positively correlated (Stevenson and Peterson, 2016; Li and Monroe, 2019), and that hope was also associated with action (Ojala, 2012a; Stevenson and Peterson, 2016; Burke et al., 2018).

A key finding that surfaced from these articles is the research on how children cope with climate change. In her articles, Ojala (2012a,b, 2013) explores three different types of coping used by children: (1) problem focused coping, which is actively trying to do something about climate change by doing something concrete, like thinking about the problem, searching for information, and directly acting upon it; (2) emotion-focused-coping, which is developing strategies to get rid of the negative emotions caused by the problem, such as de-emphasizing the threat, denial, distancing oneself from the problem through distraction and avoidance, finding social support, and hyperactivation of emotions; and (3) meaning focused coping, which is evoking positive emotions through beliefs and values while acknowledging the problem of climate change and finding meaning in a difficult situation when the problem cannot be solved at once, for example by reframing the situation in a positive manner, focusing on what is hopeful about the situation, and trusting different sources. Ojala (2012a) found that the most common coping strategy in children, adolescents and young adults was using emotional distancing from climate change (i.e., emotion-focused coping). However, when it came to promoting hope, meaning-focused coping was the most efficient coping mechanism because it activates positive emotions without ignoring the negative ones, which was associated with general positive affect and pro-environmental behavior (Ojala, 2012b). These findings were cited in other articles that also acknowledged the importance of hope in children (Stevenson and Peterson, 2016; Burke et al., 2018; Li and Monroe, 2019; Ratinen and Uusiautti, 2020; Zummo et al., 2020; Hickman et al., 2021). For example, Li and Monroe (2019) found a positive association between concern and hope in youth. This suggests that there could be a presence of strong negative emotions toward climate change in children and youth without the presence of distress, supporting the spectrum hypothesis of eco-anxiety.

Reported vulnerability factors that are associated with increased worry included the following: using problem-focused coping mechanisms (Ojala, 2013), being a girl (Stevenson and Peterson, 2016; Ratinen and Uusiautti, 2020), not having the possibility to take action (Pinto and Grove-White, 2020), having a strong connection to nature or the land, as can be seen in indigenous communities (Chalupka et al., 2020), and believing that the governmental responses are unsatisfactory (Hickman et al., 2021).

Evidence from the articles also suggested potential protective factors that could foster hope in children. For instance, having a sense of agency (Sobel, 2007; Pinto and Grove-White, 2020; Hickman et al., 2021), trusting technological advances (Strife, 2012), feeling a sense of purpose and using meaning-focused coping mechanisms (Ojala, 2012a,b, 2013), being involved in activism and having positive images of the future (Burke et al., 2018), as well as feeling empowered (Taylor and Murray, 2020) were all associated with positive management of emotions. Moreover, being a girl was associated with higher hope, and was thus also identified as a protective factor (Stevenson and Peterson, 2016; Li and Monroe, 2019). The vulnerability and protective factors by author can be found in Table 4.

### Knowledge gaps and considerations for future research

Our last objective was to identify knowledge gaps and important considerations in the literature to guide future research and help people already supporting children who are experiencing eco-anxiety. This scoping review brought to light many recommendations on how to maintain and protect the mental health of children and adolescents in the face of climate change. These recommendations are addressed to various groups of people and levels of society, including teachers and educators, parents, policy makers and political figures, school curriculum administrators, mental health professionals, fellow researchers, as well as the general public. All the recommendations by authors are also reported in Table 5. As mentioned previously, these recommendations are based on preliminary research and should be analyzed with caution. The knowledge gaps found by the authors of the included articles are highlighted as considerations for researchers.

**Table 5:** Considerations per author.

**Social agent**	**Considerations**	**Authors**
Teachers, educators	Climate action should be student-led	Pinto and Grove-White, 2020
	Promotion of realistic optimism, realistic positive thinking	Ojala, 2013; Ratinen and Uusiautti, 2020
	Consider the sociopolitical context and/or students' backgrounds when discussing climate change within the classroom	Stevenson and Peterson, 2016; Zummo et al., 2020
	Incorporate solutions into classroom discussions pertaining to climate education Give discussion opportunities to come up with ways to act, or directly give students suggestions of concrete pro-environmental actions they can take Give a sense of agency	Sobel, 2007; Ojala, 2012a, 2013; Stevenson and Peterson, 2016; Li and Monroe, 2019; Plautz, 2020; Zummo et al., 2020
	Use literature as a tool to discuss climate change with children: •Books should be carefully selected •Several resources should be used •Use books as a medium to create a dialogue, and combine this dialogue with students' perspectives and thoughts	Boggs et al., 2016
	Emphasize collective action	Ojala, 2013; Stevenson and Peterson, 2016; Pinto and Grove-White, 2020
	Give students the opportunity to engage and work with one another when addressing the issue of climate change	Ojala, 2013
	Do not hide the realities of climate change from students	Stevenson and Peterson, 2016
	Validate students' emotions, encourage students to openly discuss their feelings, consider their emotions	Ojala, 2012a, 2013
	Promote meaning-focused coping (e.g., realistic positive thinking, invite guest speaker who is actively engaged in climate change)	Ojala, 2013
	Balance encouragement of problem-focused coping with that of positive thinking, optimism, and trust in others	Ojala, 2012b
	Encourage students to seek different ideas of what the future will look like (not just from media and scientific sources, but also through art and cultural activities)	Ojala, 2012b
	Give students the opportunity to engage in environmental actions (not just discuss)	Strife, 2012
	Encourage individual and critical thinking about the environment and climate change in the following ways: •Treat learning as an educational journey, rather than imposing one's own views and values •Give students the tools and skills they need to understand and critically think about policies and ideas pertaining to the environment •Give students the chance to participate in debates	Nagel, 2005
School systems	Make changes to the school curriculum with a proposed framework called the Learn-Think-Act, while including the following: •A mental health component •Material that is age-appropriate, gender-sensitive, and intersectional •Content that involves both local and global perspectives •A focus on collectivity	Pinto and Grove-White, 2020
	Teach climate education at younger ages, rather than starting when children are older	Pinto and Grove-White, 2020
	Establish specific school-based program (i.e., Ladder of Responsibility) within schools, whereby each grade level has its own set of age-appropriate responsibilities, these tasks can require daily or weekly care or dedication on the children's part, and they are incorporated into the curriculum in various ways.	Sobel, 2007
	Encourage teaching children to engage in environmental behaviors before introducing them to knowledge that may become overwhelming and not have as much of an effect	Sobel, 2007
	Encourage nature experiences and exposure to nature; in climate education, have children engage with the environment through behavior	Sobel, 2007
Parents	Give child the space to share their concerns and emotions regarding climate change; a parent does not need to be an expert. Validate child's emotions and feelings concerning the issue Balance negative information with positive information (3 positives for each negative) Encourage child to take action, and when doing so, focus on local perspectives and tangible actions. Come up with attainable family goals to take action.	Taylor and Murray, 2020
Parents and teachers/educators	For children or students who frequently watch television, a space of conversation should be opened up by the adult, for children to openly share thoughts, feelings, and concerns	Strife, 2012
People in positions of power (e.g., politicians), adults	Take responsibility and action in the climate crisis; it should not be left to youth alone.	Hickman et al., 2021
	Demonstrate that adults are also taking care of the planet; as an adult, show care and concern	Ojala, 2013; Plautz, 2020
Macro-level, or general population	Establish a systems-based approach regarding climate change and children's mental health, with the following features: •Accessible mental health systems •Psychological first aid training •Public health surveillance and monitoring •Innovative research strategies •Environmental preservation •Social cohesion and public health through community design	Chalupka et al., 2020
	The knowledge that is passed to children should not be stretched outside of the proportion of science. Children should be receiving hopeful, yet realistic messages.	Plautz, 2020
	Provide resources and support to more vulnerable communities, as well as teach community members certain skills so that they can sustainably provide the services (e.g., psychological first aid training)	Burke et al., 2018
	Educate and engage children of Western countries in climate education, seeing that they are more distant from the realities of climate change compared to those of less developed countries, and that their actions have more of an impact (i.e., the contribute more to the causes of climate change).	Burke et al., 2018
Mental health professionals	Advocate, educate others (e.g., colleagues, decision-makers, etc.) about the impacts of climate change, as well as inform others on solutions and concrete actions that can be made	Burke et al., 2018
Researchers (future studies)	Evaluate social context	Ojala, 2012a,b
	Investigate age more closely (e.g., specific to certain age or to children in general)	Ojala, 2012b
	Examine the effects of environmental concern on immediate environmental behavior	Strife, 2012
	Explore the effects of children's concerns and feelings on long-term environmental behavior	Strife, 2012
	Examine and develop ways to build resilience in children in the face of climate change	Burke et al., 2018
	Expand research of the psychological effects of climate change on children to non-developed, non-Western, low- and middle-class countries	Burke et al., 2018
	Determine ways of encouraging children to have positive images of an attainable zero or low carbon future, as well as investigate how these images can promote mental wellbeing and healthy coping. Then with this, also determine how adults can promote these ways of coping.	Burke et al., 2018

#### Parents

Starting at the micro-level of society, even though they may not feel like they are experts in this area, parents play a significant role in their children's relationship with climate change (Taylor and Murray, 2020). In their article, Taylor and Murray (2020) give the following tips for parents when it comes to discussing and addressing the effects of climate change with their child: (1) provide the child with opportunities to openly share their emotions and concerns regarding climate change; (2) validate the child's emotions and feelings, without minimizing them; (3) balance negative information with positive information, more specifically, for each negative piece of information, give three positives; (4) when engaging younger children in environmental action, focus on the local level and on more tangible acts; and finally (5) come up with attainable goals and complete them as a family. Some of these are consistent with what other authors suggested to teachers and educators. For instance, Ojala (2012a, 2013) recommends that teachers also take the time to validate children's emotions concerning climate change, and that they too provide a safe space for students to openly share their feelings about it.

#### Teachers and educators

Much of the literature that was identified and explored highlighted several implications for teachers and educators, many of which overlapped with one another. For one, some of the authors advise that teachers make certain considerations prior to initiating discussions in class. For instance, Zummo et al. (2020) recommend that educators consider their students' backgrounds as well and the current sociopolitical context, and that they work with discourses that already exist within these contexts (especially hopeful discourse). When it comes to climate education, Stevenson and Peterson (2016) also highlight the importance of being mindful of students' diverse backgrounds, such as their socioeconomic status (SES), as they found an association between low SES and low levels of pro-environmental behaviors. These authors also suggest that education should incorporate environmental justice components to focus on environmentalism in lower SES communities who are often also the most affected by environmental degradation.

As for curriculum material itself, teachers are encouraged to use carefully selected books and literature to discuss climate change with children; they should use several resources to create a dialogue in combination with the students' own thoughts and ideas (Boggs et al., 2016). In addition, several authors recommend taking action, such as providing students with the opportunity to find and discuss concrete ways to act to improve climate (Ojala, 2013; Li and Monroe, 2019), directly giving students suggestions of concrete actions they can put into practice (Stevenson and Peterson, 2016), as well as give students hands-on opportunities to participate in environmental action (Strife, 2012). Sobel (2007) indicates that this sense of agency can lead to knowledge and motivation for environmental responsibility; and Li and Monroe (2019) mention the importance of children and adolescents feeling that they are making a difference, as they specify that environmental grief alone without any action can lead students to disengage themselves. Having this sense of agency and control over the issue can help them cope with the climate crisis, which can be a protective factor of mental health (Ojala, 2013). Moreover, teaching children to engage in environmental behaviors before introducing them to knowledge can be beneficial, since the knowledge itself may become overwhelming and not effectively promote agency (Sobel, 2007).

Several authors are specific in the types of actions they recommend, such as cycling to school, buying eco-labeled products, and composting. However, according to Pinto and Grove-White (2020), these actions should be student-led. It is also advised that teachers stress collective action, rather than individual action (Ojala, 2013; Stevenson and Peterson, 2016; Pinto and Grove-White, 2020). According to Ojala (2013), making this a collective issue rather than an individual one bears more benefits to students' wellbeing when confronting the climate crisis; giving students a space to work and engage with another to address the problem is one way in which collective action can be put into practice.

Furthermore, it is recommended that classroom discussions be solution focused; however, it is cautioned to not heavily rely on technocentrism, whereby environmental problems are solely solved by new technology. Rather, it is encouraged that other aspects also be considered like the “ethical, moral, political, and social dimensions of climate change” [(Zummo et al., 2020). p. 1222]. Plautz (2020) follows this line of thinking, as they recommend that educators not only teach children facts about climate change and environmental responsibility, but that they also focus on solutions and empowerment, to give students the sense they can change the world.

Acquiring knowledge and finding solutions to climate change are not the only goals that are proposed for climate education. Nagel (2005) encourages individual and critical thinking within the classroom, and outlines the following recommendations for environmental educators: (1) do not pre-establish any values within the classroom, instead, treat learning as an educational journey; (2) give students the tools and skills they need to understand and critically think about policies and ideas pertaining to the environment; and (3) give students the chance to participate in debates, so that they can apply these analytical skills to the information they are exposed to concerning the environment.

Finally, various articles introduce different types of coping used by students and that environmental educators should be aware of. For one, constructive hope is an important topic of investigation when it comes to environmental knowledge and awareness, as “realistic, positive expectations [hope] closely relate to self-knowledge” [(Ratinen and Uusiautti, 2020). p. 12]. It is recommended that to have this appropriate balance of environmental awareness and constructive hope, realistic optimism should be promoted, as it “helps [children] identify their own attitudes and support trust in not only themselves as environmental actors, but to mankind in general—to provide hope” [(Ratinen and Uusiautti, 2020). p. 12]. This falls in line with Ojala (2013) recommendation that teachers use meaning-focused coping in the classroom, through methods such as realistic positive thinking (as suggested by Ratinen and Uusiautti, 2020), as well as bringing in guest speakers who are engaged in the issue of climate change (e.g., scientists and activists). Problem-focused coping is another method of coping that is mentioned, whereby teachers are encouraged to balance problem-focused coping with encouraging positive thinking, optimism, and trust in others (Ojala, 2012b). For instance, teachers can invite students to seek different ideas of what the future will look like; not just from media and scientific sources, but also through art and cultural activities (Ojala, 2012b).

#### Mental health professionals

One article acknowledges the role that mental health professionals can play when it comes to children and adolescents' mental health in relation to climate change. Although brief, Burke et al. (2018) recommend that mental health professionals do the following: (1) advocate, (2) educate others (e.g., colleagues, decision-makers, etc.) about the mental health impacts of climate change, and (3) inform others on solutions and concrete actions that can be done.

#### Meso level: School systems

Pinto and Grove-White (2020) highlight that climate education is key in elementary schools, and that changes need to be made to the school curriculum to prioritize climate education. The authors recommend the addition of a mental health component to the proposed framework (i.e., the Learn-Think-Act framework), and that the content in the material that is being presented is age-appropriate, gender-sensitive, as well as intersectional. Moreover, the content itself should include both local and global perspectives. This proposed addition of climate education would involve both school-based and at-home activities, with some flexibility, beginning while children are still young, rather than when they are older, as it is argued that they are the next generation of change.

Sobel (2007) also recommends a specific program for schools called the Ladder of Responsibility. This school-based program consists of giving children tasks aimed to help the environment throughout their school years. Each grade has their own part of the “ladder,” whereby they have their own set of age-appropriate responsibilities, which becomes more challenging with time. These tasks are incorporated into the curriculum in various ways (e.g., in the science classes, but also in arts, math and social sciences through various weekly or daily activities), thus simultaneously fulfilling curriculum requirements for different subjects. Along with this, Sobel (2007) also encourages schools to expose children to nature and encourage lived experiences in nature, thus effectively engaging children with the environment through behavior.

#### Adults and people of power

According to Hickman et al. (2021), some of the literature mentions that actively participating in the fight against climate change can help with climate anxiety. However, their article suggests that in the case of children, it should not only be up to them to step up. Rather, those in power should take responsibility and action in the climate crisis by “recognizing, understanding, and validating the fears and pain of young people, acknowledging their rights and placing them at the center of policy making.” (p. 9). Ojala (2013) and Plautz (2020) support this need for adult accountability, as they encourage adults who surround children to show them that they also care about this pressing issue, and that they too are taking action in caring for the planet.

Adults also hold the responsibility of communicating knowledge and information relating to climate change in a certain manner. For one, the information that is being communicated should not be given to children in a way such that it is stretched outside of the proportion of science; rather, children should be receiving hopeful, yet realistic messages (Plautz, 2020). With this, adolescents should be exposed to the realities of climate change, and adults should not hide these realities from them. Although it is necessary to ensure that hope is not lost and that adolescents do not get discouraged, Stevenson and Peterson (2016) argues that adolescents are able to react to these realities in an action-driven way when given the appropriate tools.

#### Macro level and the general population

Chalupka et al. (2020) proposes a systems-based approach when it comes to climate change and children's mental health, which includes the following: (1) accessible mental health systems, (2) psychological first aid training, (3) public health surveillance and monitoring, (4) innovative research strategies, (5) environmental preservation, (6) social cohesion and public health through community design. All these measures are aimed to promote children's mental health during the climate crisis. Burke et al. (2018) also focus on the need for psychological support; however, they further recommend that such resources and support be provided to more vulnerable communities. Yet at the same time, they state that children from Western countries should especially engage in climate education and action, as they are more distant from the realities of climate change compared to those of less “developed” countries, and their actions have more of an impact (i.e., they contribute more to the causes of climate change).

Similar to Burke et al. (2018) and Chalupka et al. (2020) also recommend the use of community design, whereby community members are taught certain skills so that they can sustainably provide such services (e.g., psychological first aid training).

#### Researchers: Suggestions for future research

With the little empirical evidence that was gathered in this scoping review, it is to no surprise that a variety of future routes of research were proposed. Burke et al. (2018) suggest two practice-focused areas. Firstly, future research could examine and develop ways to build resilience in children in the face of climate change. Secondly, future studies could determine ways of encouraging children to have positive images of an attainable zero or low carbon future, as well as investigating how these images can promote mental wellbeing and healthy coping, then determining how adults can promote these ways of coping. The need for the expansion of research on the psychological effects of climate change on children to “non-developed,” non-Western, low- and middle-class countries is also encouraged.

Ojala (2012a,b) brings up the need for certain variables and factors to be more closely considered. For instance, they mention that age needs to be investigated in a more specific manner (e.g., are these findings specific to 12-year-olds or are they found in other age groups as well?). They also acknowledge the importance that future studies examine context more closely when researching coping. Lastly, Strife (2012) presents two potential topics that can be used in future research, namely the effects of environmental concern on immediate environmental behavior, as well as the effects of children's concerns and feelings on long-term environmental behavior.

## Discussion

The present scoping review included 18 articles that discussed the presence of vicarious reactions to climate change in children. The aim of this study was to explore the terminology used to describe eco-anxiety, to look at the evidence of eco-anxiety in children, and to identify the knowledge gaps and considerations for future research in this research topic.

### Terminology used

The present results highlight an irregularity in the terminology used to describe children's emotional reaction to the awareness of climate change. Very few articles used the term eco-anxiety (16%), and those that did had different definitions of it; although, worry was a commonality amongst them. Other terms were also used to describe a fear and worry of environmental consequences in children including ecophobia, climate anxiety, environmental grief, and eco-despair. This lack of clarity is consistent with previous literature that finds that there are many different terms used that overlap in their definition, suggesting a need to be further investigated (Coffey et al., 2021). This variability of terms observed in the literature is representative of a developing area of research and a complex situation that is still being defined (Pihkala, 2020). Nonetheless, the most common word found in 14 of the articles was worry. The present review tends to confirm that, in children, eco-anxiety seems to manifest itself as many different emotions, not only fear, as the APA definition would suggest (see Clayton et al., 2017). Finally, none of the selected articles focused on a child-specific definition of eco-anxiety, rather applying those that emerged from literature in adults. Future research could aim to evaluate if eco-anxiety should be conceived and/or defined in the same way in children and adults alike. This is particularly important because applying an adult-centric definition to children may impose emotions on them and fail to acknowledge their reality. For example, research on anxiety disorders has demonstrated that the clinical manifestations of anxiety in children are different than in adults, and this could be the case with eco-anxiety (Beesdo et al., 2009)). Given that the definition is closely linked to the symptoms of anxiety, it is essential that care is taken to adequately explore these experiences to guide a definition that closely resembles children's reality. Initial evidence seems to suggest that children express a range of emotions that are often related to objects or people (Strife, 2012), whereas adults experience may be more existential in nature (Pihkala, 2020).

### Evidence of eco-anxiety in children

Variability was also observed in the emotions related to the awareness of climate change, including fear, anger, hopelessness, worry, hope, and sadness. These emotions could potentially constitute different expressions of eco-anxiety in children, as it has been seen in adults (Arcanjo, 2019). However, some authors found that eco-anger, eco-anxiety, and eco-depression in adults were all related but different constructs (Stanley et al., 2021). It can be hypothesized that this would be the case in children, but none of the articles from the current review had a child- or youth-specific measure of eco-anxiety, rather they measured worry or climate-related emotions.

Our results support that eco-anxiety, and its variations, don't constitute a pathological problem. However, the included articles did not explore the association between anxiety or depressive disorders and eco-anxiety, which constitutes an important gap in knowledge. Nonetheless, many of the authors mention that these emotions should not be considered pathological (Ojala, 2012a; Strife, 2012; Hickman et al., 2021). Authors Doherty et al. (2017) explained that, in adults, potential pathology associated with eco-anxiety is very context-dependent and case specific, so eco-anxiety should not be considered as a psychological problem from the outset. These same conclusions are found in the results of the present review, where eco-anxiety and eco-emotions may be positive reactions, in that they may lead to action. This could also lend support to the spectrum hypothesis of eco-anxiety, where on the one hand, children who experience strong emotions and who cope in a positive manner may be more hopeful and act (Ojala, 2012a; Burke et al., 2018; Taylor and Murray, 2020). On the other hand, some children may be overwhelmed by these feelings, and lack the tools to properly cope, leading to potential paralysis, learned hopelessness, and denial (Nagel, 2005). This spectrum interpretation of eco-anxiety should be further investigated and adapted to children and their specific contexts.

The vulnerability factors found in the present review are consistent with the growing body of literature that indicates that young people, indigenous groups, and people who feel connected to nature are particularly vulnerable to experiencing eco-anxiety and particular mitigation measures should be put into place to protect these groups from experiencing further trauma (Coffey et al., 2021). These vulnerability factors should be taken in account when further investigating the phenomenon of eco-anxiety in children as well as in education contexts.

Interestingly, the present scoping review revealed that girls were both more likely to feel worried and hopeful regarding climate change. This is concordant with previous research that reveals that girls are at higher risk of feeling internalizing symptoms, which include feeling sad, anxious, nervous, irritable, and depressed (Merrell and Dobmeyer, 1996; Bor et al., 2014). However, this may occur because girls generally develop higher levels of emotional awareness (Eastabrook et al., 2014), which would explain why they would also report higher levels of hope. Research with adults also confirm the women tend to have higher levels of climate change related worry (Heeren et al., 2021) and that this is potentially mediated by risk perception (Xiao and McCright, 2012). These gender differences warrant to be further investigated as there is also research finding no gender differences in worry (Clayton and Karazsia, 2020) and that gender is often measured in a binary manner.

### Considerations and future research

Interestingly, many recommendations for specific social agents in children's lives surrounding the topic of eco-anxiety emerged; however, the novelty of the field and the need for more rigorous methodologies commends the importance of further research. Nonetheless, these preliminary considerations are important to guide researchers, parents, teachers and educators, mental health professionals, and people of power who have concerns about supporting children in the context of the climate crisis.

#### Parents

It is important for parents to acknowledge that their children will be learning about climate change through school, the media, and the internet, so their role is to maintain an open discussion with their children and adolescents, creating a safe space to discuss feelings and emotions about the issue (Strife, 2012). Books may be an excellent means of initiating conversations and tackling the issue of climate change with age-appropriate material (Boggs et al., 2016). Local bookstores can be helpful in identifying these books. Furthermore, the available scientific literature indicates that children imitate their parents' environmental behaviors and take on their values (Zerinou et al., 2020). Thus, it is important that parents be aware of their role as models to encourage environmental action that can provide solace for children who may be particularly concerned about climate change. An important gap in research exists in how parents' reactions and behaviors may affect how their children cope with climate change. For example, to what extent does having a parent who experiences eco-anxiety affect a child's emotional response to climate change? Future research should focus on the specific experiences of families that vicariously experience climate change.

#### Teachers and educators

Results from this scoping review indicate that teachers and educators should first teach their students environmental behaviors before introducing them to the facts about climate change (Sobel, 2007). Indeed, if children already have the tools to feel agency in the face of climate change, when they start learning about it, they may not feel hopeless and distressed. However, caution is warranted in excessively using action as an “antidote to eco-anxiety,” as it can lead to burnouts or an unproportionate importance of individual action over collective, governmental, and large polluters' action (Pihkala, 2020). A significant research gap exists in how to avoid this type of reaction in children. Classrooms should also provide opportunities for students to normalize their emotions of guilt, sadness, or anger through group discussions (Plautz, 2020). Similar to what Ojala (2012a) has suggested, other authors support the importance of building resilience to cope with climate change, for example reframing the problem of climate change by highlighting the positive opportunities, fostering meaning-focused coping (Baudon and Jachens, 2021). Finally, teachers should also empower their students and teach them critical thinking skills, for example, to recognize reliable information (Nagel, 2005). For instance, philosophy for children workshops could provide a space for children to discuss the issue of climate change while enabling them to think by and for themselves (Birch, 2020). However, teachers and educators may not feel like they have the adequate tools to put these into practice, so it is important that future research focuses on the needs of instructors to provide them with accessible tools to support their practice and their students. A few researchers have suggested ideas to support a change in education systems to integrate notions of collective actions moving away from simple climate change literacy (for example, González-Gaudiano and Meira-Cartea, 2019), but these new educational practices should also be further investigated in different contexts.

#### Mental health professionals

Very little indications arise for mental health professionals specifically, although some of the aforementioned suggestions may be applicable in a clinical context. Literature indicates that psychology can contribute to policies, prepare communities for the impacts of climate change, and individually help their clients cope with the potential distress (Berry, 2009; Baudon and Jachens, 2021). However, future research is required to identify potential interventions to help children develop coping mechanisms to deal with the awareness of climate change, and child or youth-specific mental health interventions to reduce eco-anxiety when there is. Furthermore, psychologists and mental health workers may benefits from training to specifically help clients who are experiencing distressing eco-anxiety (Swim et al., 2011; Pinsky et al., 2020).

#### School systems

Climate education and specific programs should be implemented in schools that also support children and youth's mental health (Pinto and Grove-White, 2020). Authors included in this review also suggest introducing climate education at the beginning of elementary school, including attainable actions per grade, and incorporating exposure to nature (Sobel, 2007; Pinto and Grove-White, 2020). This component of nature was not further explored in any of the selected articles; although, child exposure to nature could predict adult environmental attitudes and behavior, and nature-based therapies have been beneficial to help adults with their eco-anxiety (Asah et al., 2018; Baudon and Jachens, 2021). Given that nature exposure could help adults reduce their depressive symptoms (Watkins-Martin et al., 2021), future school-based programs should explore the benefits of integrating an outdoor component that could enhance children and youth's connection to nature (Collado and Corraliza, 2015; Malboeuf-Hurtubise et al., 2022). However, it will be important that upcoming research compare the effects of the different school programs to understand what strategies work best, and especially how these could support children and youth mental health as they learn about the consequences of climate change.

#### Adults and people of power

It is recommended that people who are in power acknowledge their responsibility in the climate crisis (Hickman et al., 2021; Pickard, 2021). Taking off the burden of the duty to mitigate climate change that has been put on the shoulders of youth and children and showing them that greater action is being taken by governments can potentially reduce the distress felt by this generation (Hickman et al., 2021), although this remains to be further investigated. Adults in general need to be accountable and act in accordance, to show young people they are stepping up and caring for the planet (Ojala, 2013; Plautz, 2020). Furthermore, children and youth should be consulted in decision-making and further encouraged to have a voice in community affairs; however, the ways in which to do this successfully should be further investigated (Vogiatzi et al., 2017).

#### Macro level: General population

Communities also have the responsibility to protect children and youth's mental health by preparing for potential environmental disasters (i.e., floods, fires, etc.) (Burke et al., 2018). It is important that more vulnerable communities, because of their geographic location or socioeconomic status, be especially supported in the development of different approaches to support mental health. Indeed, building resilience to climate change may be essential, but little literature exists on how this may be achieved and how to tailor such preventive strategies to different communities around the world (Chen et al., 2020).

#### General gaps in research

The current review only identified studies employing research designs that were descriptive in nature, indicating a gap in empirically based data using rigorous methodological designs. Indeed, future research could move toward inferential designs to explore the causality between certain variables associated to eco-anxiety in children, such as coping mechanisms, emotional resilience, and general knowledge about climate change, to name a few.

In general, research on eco-anxiety seems to be moving much slower than the public discourse on the topic (Pihkala, 2020), especially when it comes to children. This review puts to light the importance of better defining the concept of eco-anxiety in children, using research methods that could give children and youth a voice in their experience, as suggested by Strife (2012). Furthermore, gaps exist in the specific experience of children and youth who are already living with a mental health problem, and how they are specifically affected by the awareness of climate change. Hickman et al. (2021) suggest that “A complete understanding of climate anxiety in children and young people must encompass relational, psychosocial, cultural, ethical, legal, and political factors” (p. 9).

#### Strengths and limitations

This scoping review presents multiple strengths. First, this review follows the strict methodological guidelines suggested by Levac et al. (2010) and Tricco et al. (2018) for conducting rigorous scoping reviews. Some limitations are inherent to scoping reviews, such as providing breadth rather than depth of knowledge in the subject (Tricco et al., 2018). This can be specifically seen in the multiple databases searched. However, given that the topic of eco-anxiety is interdisciplinary, emerging, and that the concept in children warranted to be clarified, this method was appropriate. Nonetheless, we suggest that, as research on eco-anxiety continues to emerge, future studies should go in depth into the articles and evaluate the quality of these. Furthermore, to our knowledge, only one other scoping review (Martin et al., 2021) was published on the topic of eco-anxiety in children, thus our work helps in laying the groundwork for future research and questions on this important topic.

In terms of limitations, both reviewers responsible for evaluating the relevance and summarizing sources included in this scoping review had a psychology background. However, great care was taken to be aware of potential biases and remaining neutral in the inclusion of the articles. Furthermore, no books, nor literature in other languages that English were included (no relevant French material was found), leading to potentially important data to be missed.

Finally, we acknowledge that an important, yet still small, body of literature that focuses on the experience of indigenous communities' experiences of climate change was not included in the present review given that the articles found did not focus on children's experiences. This decision was also made because these communities often already experience the direct and indirect effects of climate change, so vicarious mental health effects seem less present (or, at least, less documented in the available literature). Nonetheless, indigenous communities have unique challenges that should not be ignored in this line of research (please see Hunter, 2009; Cunsolo et al., 2013; MacKay et al., 2020; Middleton et al., 2020).

## Conclusion

This scoping review highlighted the presence of eco-anxiety in children and youth. Indeed, this population experiences a variety of emotions such as anger, sadness, guilt, and hopelessness that characterize eco-anxiety. However, none of the included articles had child-specific measures of this concept, suggesting that future research should further investigate the phenomenon from a child-specific perspective. This review also underlines the important roles of parents, teachers/educators, mental health professionals, school systems, and adults and people of power to mitigate the effects of climate change on children and youth's mental health. Although the identified research lays the groundwork for the topic of eco-anxiety in children, many research gaps are highlighted as future directions for research.

## Data Availability Statement

The original contributions presented in the study are included in the article/supplementary material, further inquiries can be directed to the corresponding author.

## Author contributions

Conceptualization: TL-G. Methodology, formal analysis, and writing–original draft of manuscript: TL-G and TM. Writing–review and editing: CM-H, MG, and P-OP. Supervision: CM-H and CC. All authors have read and agreed to the published version of this manuscript.

## Funding

The main researcher, TL-G was funded for her master's degree by the Fond de Recherche du Québec –Société et culture (FRQSC) (Dossier #B1Z-301502). CM-H, CC, and TL-G also received a Social Sciences and Humanities Research Council of Canada (SSHRC) Connection Grant for this scoping review (reference number: 611-2021-0199).

## Contribution to the field statement

Given the limited research on eco-anxiety and youth, this scoping review lays the groundwork for future research directions on this topic. Specifically, it helps identify key elements from what is currently known in the literature, such as the importance of promoting hope to enable action. Furthermore, it identifies the research gaps and raises important research questions, including how to help youth cope with eco-anxiety. This review can inform the work of policymakers, activists and mental health workers who undoubtedly will be called upon to find solutions for the kids living with eco-anxiety.

## Conflict of interest

The authors declare that the research was conducted in the absence of any commercial or financial relationships that could be construed as a potential conflict of interest.

## Publisher's note

All claims expressed in this article are solely those of the authors and do not necessarily represent those of their affiliated organizations, or those of the publisher, the editors and the reviewers. Any product that may be evaluated in this article, or claim that may be made by its manufacturer, is not guaranteed or endorsed by the publisher.

## Figures and Tables

**Table:** 

**Author(s), title, year**	
Country	
Study design, participants	
Age mentioned	
Study aims, measures, main impacts	
Definition of eco-anxiety or related concept, other terminology used	
Impact of eco-anxiety on children	
Vulnerability and protective factors	
Author recommendations	

## Appendix

**Appendix A: Search strategy example**

Step 1: An initial limited search was done in APA PsycInfo on March 4^th^, 2021

Step 2: Keywords for each key concepts included:

Box 1 **Search Term #1:** ABSTRACT(Child* OR Kid OR Adolescen* OR Youth OR Teen* OR Preschool* OR Kindergarten* OR School-aged OR Elementary OR School OR parent* OR famil*)

AND

Box 2 **Search Term #2:** ABSTRACT(“Global warming” OR “Climate change” OR “Ecological crisis” OR “Climate disaster” OR “Environmental disaster” OR “Environmental problems” OR “climate crisis” OR “environmental melancholia” OR “ecological disaster” OR “Environmental Issues”)

AND

Box 3 **Search Term #3:** ABSTRACT(“Eco-anxiety” OR Ecoanxiety OR solastalgia OR “ecological worry*” OR Angst OR “Mental health” OR Anxiety OR Depression OR Hope* OR Anticipatory OR Despair OR Optimism OR Engagement OR Emotions OR Grief OR Ecophobia OR attitudes OR “Psychological effects” OR worry OR resilience OR “eco-psychology” OR “eco-angst” OR “eco-guilt” OR “eco-paralysis” OR “environmental concern” OR “climate activism”)

This initial search yielded 284 articles.

**Appendix B: Scoping review protocol**

**Review title:** Eco-anxiety in Children: A scoping Review of the Mental Health Impacts of the Awareness of Climate Change

**Review question:** What is the nature of the evidence on eco-anxiety in youth and children?

**Inclusion criteria (PCC):**

Population– Children (under 18 years of age)

Concept–Eco-anxiety: “a general term for difficult feelings because of the ecological crisis”

Context–Climate change

**Exclusion criteria:**

Studies that only included mental health impacts of climate related events (e.g., after a hurricane) were not included.

Only material in English and in French were examined due to the language capacity of the reviewers.

**Study details and characteristics extraction:**

Initial data chart
